# Hyperdense middle cerebral artery sign in large cerebral infarction

**DOI:** 10.1002/brb3.2116

**Published:** 2021-03-25

**Authors:** Jie Hou, Yu Sun, Yang Duan, Libo Zhang, Dengxiang Xing, Xiaoqiu Lee, Benqiang Yang

**Affiliations:** ^1^ Department of Radiology General Hospital of Northern Theater Command Shenyang China; ^2^ Department of Radiology Center for Neuroimaging General Hospital of Northern Theater Command Shenyang China; ^3^ Department of Medicine Data General Hospital of Northern Theater Command Shenyang China; ^4^ Department of Neurology General Hospital of Northern Theater Command Shenyang China

**Keywords:** atrial fibrillation_3_, computed tomography_5_, hemorrhagic transformation_4_, hyperdense middle cerebral artery sign_1_, large cerebral infarction_2_

## Abstract

**Objectives:**

To evaluate if the hyperdense middle cerebral artery sign (HMCAS) is an imaging biomarker for hemorrhagic transformation (HT) and the functional outcome of patients with large cerebral infarctions without thrombolytic therapy.

**Materials and Methods:**

The clinical and imaging data of 312 patients with large cerebral infarction without thrombolytic therapy were retrospectively analyzed. They were divided into patients who presented with HMCAS (*n* = 121) and those who did not (non‐HMCAS[*n* = 168] patients), and the clinical data of the 2 groups were compared. This was a retrospective study.

**Results:**

Of the 289 patients, 83(28.7%) developed HT. The incidence of atrial fibrillation, high homocysteine and admission NIHSS score at the time of admission was significantly higher in the HMCAS patients than in non‐HMCAS patients (*p* < .05). The ASPECTS was significantly lower in HMCAS patients (*t* = −5.835, *p *< .001). The incidence of PH‐2 and 3‐month mRS score was also statistically significant higher in HMCAS patients (χ^2^ = 3.971, *p* = .046; χ^2^ = 5.653, *p *< .001, respectively). A sub‐analysis showed HMCAS patients with HT were significantly older than non‐HMCAS patients with HT (*t *= 2.473, *p* = .015). The incidence of atrial fibrillation and the 3‐month mortality rate were higher in HMCAS patients with HT than in non‐HMCAS patients with HT (χ^2^ = 3.944, *p* = .047; χ^2^ = 6.043, *p* = .014, respectively). Multiple logistic regression analysis showed HT was independently associated with HMCAS (adjusted OR/95% CI/*p* = 2.762/1.571–4.854/*p *< .001) and admission NIHSS score (adjusted OR/95% CI/*p* = 1.081/1.026–1.139/0.003). And HMCAS with HT was independently associated with length of HMCAS (adjusted OR/95% CI/*p* = 1.216/1.076–1.374/0.002).

**Conclusions:**

HMCAS in patients with a large cerebral infarction without thrombolytic therapy is an independent biomarker of HT. Length of HMCAS is also a marker of HT with lower ASPECTS in HMCAS patients.

## Graphical abstract

Hyperdense middle cerebral artery sign (HMCAS) in patients with a large cerebral infarction without thrombolytic therapy is an independent marker of hemorrhagic transformation (HT). Length of HMCAS is also a marker of HT with lower ALberta Stroke Program Early CT score (ASPECTS) in HMCAS patients.

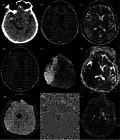

## 1 INTRODUCTION

Imaging biomarker is increasingly used to provide a better understanding of the pathophysiology of acute ischemic stroke and helps to improve the development of novel treatment strategies (Harston et al., 2015). Computed tomography (CT) and magnetic resonance imaging (MRI) play a central role in the management of acute stroke. CT perfusion (CTP), diffusion‐weighted imaging (DWI) could be useful in guiding reperfusion therapy in acute ischemic stroke patients (Katyal & Bhaskar, 2020). The hyperdense middle cerebral artery sign (HMCAS) is one of the consistently recognized CT imaging signs of acute ischemic stroke (Mair et al., 2016). Although HMCAS on the admission CT has been well established as an early biomarker of thromboembolic arterial occlusion (Jensen et al., 2010), when facing all kinds of treatment, large cerebral infarction with HMCAS needs to be further studied.

Thrombolytic therapy is the first‐line treatment for acute ischemic stroke. Stroke patients are treated with intravenous thrombolytics within the first 3 or 4.5 hr after onset of symptoms (Hacke et al., 2008). Unfortunately, only 11% of all stroke patients are deemed eligible to receive thrombolytic therapy (Mair & Wardlaw, 2014). Recently implemented interventions such as mechanical embolectomy or direct intra‐arterial delivery of a thrombolytic agent also show potential for recanalizing the blood vessel in cases of arterial occlusion shown on angiography (Broderick et al., 2013; Kidwell et al., 2013; Mair & Wardlaw, 2014). However, patients with large cerebral infarction need to be evaluated before treatment, and their clinical prognosis should be predicted.

Hemorrhagic transformation (HT) is a serious complication secondary to large cerebral infarctions, which has a poor prognosis, including death (Jickling et al., 2014). HMCAS may provide useful information to clinicians permitting early intervention. Clinicians, including neurologists, have remained unsure about HT and the outcomes of patients with large cerebral infarctions showing HMCAS without thrombolytic therapy. The aims of this retrospective study were to assess the incidence and baseline characteristics of patients with HMCAS on admission CT, and to determine if the HMCAS is an accurate imaging biomarker for addressing HT and the functional outcome of patients with large cerebral infarctions who do not receive thrombolytic therapy.

## 2 MATERIALS AND METHODS

The study was approved by the local ethics committee. All patients or their families provided written informed consent.

### 2.1 Patient selection

Patients with large cerebral infarctions admitted to our hospital from January 2016 to July 2020 were selected in this study. Of the total of 457 patients who were evaluated, 168 patients who did not match the inclusion criteria were excluded from our study, and 289 patients were considered eligible and were enrolled in the study. The inclusion criteria were as follows: (a) onset of signs/symptoms based on international criteria for the diagnosis of stroke (European Stroke Organisation (ESO) Executive Committee; ESO Writing Committee (2008)), (b) hospital admission within 24 hr after onset of signs/symptoms, (c) infarction lesions could not be seen on the first noncontrast‐enhanced CT examination (performed on admission prior to treatment), (d) brain imaging (a follow‐up CT and MRI) were performed within 24 hr after stroke onset showed with large infarcts (infarction area ≥ 20 cm^2^ or lesions involving more than 2 lobes) (Arsava et al., 2017), and (e) during the follow‐up period, 157 patients received CT re‐examination and 132 patients received MRI re‐examination between 24 hr and 3 weeks demonstrated infarcts with/without bleeding. The exclusion criteria were as follows: (a) patient underwent thrombolytic therapy, (b) brain trauma and tumor, (c) past history of large infarction, (d) calcification in the middle cerebral artery on CT and increased hematocrit (Abul‐Kasim et al., 2009), (e) allergy to contrast media, (f) patients with stroke of the posterior circulation infarction were excluded in our study.

### 2.2 Clinical treatment

Although the early clinical signs/symptoms of the 289 patients were consistent with the criteria for stroke, thrombolytic treatment was not administered, because they did not have the indications for undergoing thrombolysis (Powers et al., 2019; Torbey et al., 2015). The treatment for these patients was primarily to control symptoms and support the patient.

Reasons Patients Did Not Undergo Thrombolytic Therapy: (a) 185 ischemic stroke patients who awoke with time last known to be at baseline state over 6 hr; (b) 46 patients with history of thrombocytopenia, infective endocarditis, aortic arch dissection, intra‐axial intracranial neoplasm; (c) 21 patients with recent severe head trauma; (d) 13 patients with history of intracranial or spinal surgery within the prior 3 months; (e) 17 patients with blood pressure > 185/110 mmHg; (f) 7 patients with gastrointestinal bleeding within 3 weeks.

### 2.3 Neuroimaging acquisition and analysis

(1) Imaging was performed by a 256‐slice multidetector (MD) CT scanner (CT 750 Discovery HD, General Electric Company). The following imaging parameters were used: tube voltage = 120 kV, tube current = 264 mAs, slice thickness 5 mm, field of view (FOV) 240 × 240, and matrix 512 × 512.

(2) MRI was performed by a 3.0‐T scanner (Discover 750; General Electric Company). MRI acquisitions were performed with the following parameters: T1 weighted imaging(T1WI): FOV 24 × 24 cm, slice thickness 6 mm, matrix 320 × 19, repetition time (RT)]/echo time(ET)/inversion time (IT) 1,625 ms/24 ms/720 ms); T2 weighted imaging(T2WI) fast spin echo sequence; FOV 24 × 24 cm, slice thickness 6 mm, RT/ET 4,160 ms/88 ms, matrix 320 × 320); Fluid‐attenuated inversion recovery(FLAIR) sequence: FOV 24 × 24 cm, slice thickness 6 mm, matrix 320 × 192, RT/ET/IT 8,000 ms/165 ms/2,000 ms, layer spacing 1.2 mm.

For additional assessments of the infarction, the following MR data and procedures were produced, as follows: DWI spin echo sequence: FOV 24 × 24 cm, slice thickness 6 mm, matrix 128 × 128, RT/ET 4,000 ms/68 ms, layer spacing 1.2 mm, number of excitations per step (NEX) 1; Three‐dimensional time of flight Magnetic resonance angiography (3D TOF MRA); FOV 24 × 24 cm, matrix 352 × 256, slice thickness 0.6 mm, RT/ET Min ms/2.3 ms. To assess HT, the following MR procedure was performed: susceptibility‐weighted imaging (SWI): FOV 24 × 24 cm, matrix 320 × 320, layer thickness 1 mm, RT/ET 40 ms/25 ms.

HMCAS can be defined qualitatively as any artery that appears denser than adjacent or equivalent contralateral arteries (Jha & Kothari, 2009; Mullins, 2005); however quantitative definitions (>43 Hounsfield units [HU] or > 1.2‐fold the density of a normal contralateral vessel) have been proposed (Koo et al., 2000; Mair & Wardlaw, 2014). For objective evaluation of HMCAS, CT attenuation in HU on axial images by placing oval or elliptical region of interest over MCA on both sides. If MCA attenuation > 43 HU and > 1.2‐fold the normal contralateral vessel attenuation, the affected side was defined where HMCAS were subjectively seen compared to the healthy side (Abul‐Kasim et al., 2009). All image variables were measured independently by two observers (two neuroradiologists) who were blinded to the symptom or the side affected. In cases of disagreement between two observers, they settled by consensus. And the length of HMCAS and other numerical analysis were taken as the mean value between the two independent neuroradiologists.

Baseline variables included symptom onset to hospital, onset to imaging, and onset to treatment time (as the time from stroke onset to treatment after admission), age, sex, number of stroke risk factors, hypertension (blood pressure at admission), diabetes mellitus (glucose value at admission), atrial fibrillation, high homocysteine (homocysteine level at admission) and admission National Institute of Health Stroke Scale (NIHSS) score (Kwah & Diong, 2014). The extent of ischemic change using the Alberta Stroke Program Early CT score (ASPECTS) (Barber et al., 2000) of diagnostic infarction images was recorded. This score evaluates ten anatomic sites within the MCA territory for signs of ischemic change and produces a normal maximum score of 10 (no ischemic changes), minus one point for each area with ischemic changes. All variables were evaluated blinded to outcome and clinical data. Discordant opinions were settled by consensus.

### 2.4 Clinical outcomes and follow‐up

Three months after symptom onset, the patient's functional outcomes were assessed using a modified Rankin Scale (mRS) by telephone interview. The interviewers were blinded to prognostic factors during the follow‐up evaluation and were trained to administer a standard telephone interview protocol.

### 2.5 Statistical analysis

Data were analyzed using SPSS v. 20.0 (SPSS Statistics, IBM Corporation, Armonk, New York). Mean ± *SD* was used to express measurement data, and the continuous variables were analyzed by the independent sample *t*‐test (Tables 1, 2, 3). Pearson's chi‐squared test was used to assess categorical data (Tables 1, 2, 3). The degree of interobserver agreement in the subjective evaluation regarding the occurrence of HMCAS was evaluated by cross tabulation and calculation of kappa (κ values). A kappa of one indicates complete agreement of all observes in all cases whereas a kappa of zero indicates that any observed agreement is attributed to chance. Multivariate logistic regression analysis was used to identify significant independent predictors (Tables 4 and 5). Univariate and multivariate logistic regression analysis was applied for the binary outcomes with HT and without HT. And multi‐collinearity analyses were used to see if any of the covariates were collinear. Variables in Table 4 include age, gender, blood pressure at admission, glucose value at admission, atrial fibrillation, homocysteine level at admission, prestroke medications, HMCAS and admission NIHSS score. Variables in Table 5 include age, gender, blood pressure at admission, glucose value at admission, atrial fibrillation, homocysteine level at admission, prestroke medications, length of HMCAS and admission NIHSS score. Results are expressed as unadjusted or adjusted odds ratios with corresponding 95% CI. All probability values were 2‐sided and a *p*‐value < .05 was considered statistically significant. The receiver operating characteristic (ROC) analyses were used to evaluate the predictive potential in a multivariable‐adjusted logistic regression model.

**TABLE 1:** Baseline characteristics of patients with/without HMCAS

	With HMCAS (*n* = 121)	Without HMCAS (*n* = 168)	*t* or χ^2^	*p*
Mean age ± *SD*, years	67.1 ± 10.7	65.8 ± 11.1	0.972	.332
Sex (Male), *n* (%)	79 (65.3)	120 (71.4)	1.236	.266
Hypertension, *n* (%)	73 (60.3)	96 (57.1)	0.294	.587
Blood pressure at admission (mmHg)				
Systolic pressure at admission (mmHg)	144.0 ± 26.9	143.5 ± 24.9	0.169	.866
Diastolic pressure at admission (mmHg)	83.2 ± 13.1	85.4 ± 14.7	−1.327	.186
Diabetes mellitus, *n* (%)	36 (29.8)	50 (29.8)	0.000	.999
Glucose value at admission (mmol/L)	6.5 ± 3.1	6.2 ± 2.0	1.040	.299
Atrial fibrillation, *n* (%)	47 (38.8)	38 (22.6)	8.918	.003*
High homocysteine at admission, *n* (%)	24 (19.8)	18 (10.7)	4.711	.030*
Homocysteine level at admission (umol/L)	15.5 ± 10.3	13.6 ± 6.7	1.974	.049*
Prestroke medications				
Antiplatelets	37 (30.6)	42 (25)	1.102	.294
Anticoagulation	28 (23.1)	31 (18.5)	0.952	.329
Antihypertensive	68 (56.2)	87 (51.8)	0.551	.458
Anticholesterol	27 (22.3)	43 (25.6)	0.413	.521
Median onset to treatment time, min (IQR)	392 (301.5–480)	396 (310–530)	−1.096	.274
Median onset to imaging time, min (IQR)	380 (285.5–461.5)	382 (300–518)	−1.205	.229
Median onset to hospital time, min (IQR)	367 (270–450)	371.5 (290–506)	−1.403	.162
Mean length of HMCAS (mm) (IQR)	18.3 (13.5–22.6)			
Admission NIHSS score, points, median (IQR)	16 (13–21)	13 (9–16)	5.725	<.001*

Abbreviations: HMCAS, hyperdense middle cerebral artery sign; IQR, interquartile range; *n*, number; NIHSS, National Institutes of Health Stroke Scale; *SD*, standard deviation.

* Indicates statistical significance.

**TABLE 2:** Follow‐up imaging data and clinical data of patients with/without HMCAS

	With HMCAS (*n* = 121)	Without HMCAS (*n* = 168)	*t* or χ^2^	*p*
Location of occlusion by MRA, *n* (%)				
M1 MCA	74 (61.2)	81 (48.2)	4.738	.030*
M2 MCA	12 (9.9)	25 (14.9)	1.552	.213
M3 MCA	5 (4.1)	12 (7.1)	1.152	.283
TICA	11 (9.1)	23 (13.7)	1.434	.231
ICA + MCA	19 (15.7)	27 (16.1)	0.007	.933
ASPECTS, median (IQR)	5 (2–7)	6 (5–7)	−5.835	<.001*
Hemorrhagic transformation	53 (43.8)	30 (16.8)	23.128	<.001*
HI−1	8 (15.1)	7 (23.3)	0.878	.349
HI−2	9 (16.9)	6 (20)	0.118	.731
PH−1	10 (18.9)	9 (30)	1.345	.246
PH−2	26 (49.1)	8 (26.7)	3.971	.046*
Discharge NIHSS score, points, median (IQR)	8 (5–14)	5 (3–10)	5.08	<.001*
3‐month mRS score, grade, median (IQR)	4 (2–6)	2 (1–4)	5.653	<.001*
Mortality 3‐month follow‐up	38 (31.4)	20 (11.9)	16.674	<.001*

Abbreviations: ASPECTS, Alberta Stroke Program Early CT score; HI‐1, Hemorrhagic infarction type 1; HMCAS, hyperdense middle cerebral artery sign; ICA, internal carotid artery; IQR, interquartile range; MCA, middle cerebral artery; mRS, modified Rankin Scale; *n*, number; NIHSS, National Institutes of Health Stroke Scale; PH‐1, Parenchymal hematoma type 1; TICA, terminal internal carotid artery.

* Indicates statistical significance.

**TABLE 3:** Clinical and imaging data of patients with secondary HT

	With HMCAS (*n* = 53)	Without HMCAS (*n* = 30)	*t* or χ^2^	*p*
Mean age ± *SD*, years	70.0 ± 11.3	63.1 ± 13.3	2.473	.015*
Sex (M), *n* (%)	31 (58.5)	19 (63.3)	0.188	.665
Hypertension, *n* (%)	30 (56.6)	22 (73.3)	2.291	.130
Systolic pressure at admission (mmHg)	144.8 ± 29.1	149.8 ± 22.2	−0.828	.410
Diastolic pressure at admission (mmHg)	83.8 ± 12.0	85.9 ± 16.3	−0.654	.515
Diabetes mellitus, *n* (%)	13 (24.5)	8 (26.7)	0.046	.830
Glucose value at admission (mmol/L)	6.23 ± 3.27	5.81 ± 1.11	0.684	.496
Atrial fibrillation, *n* (%)	24(45.3)	7 (23.3)	3.944	.047*
High homocysteine, *n* (%)	8 (15.1)	5 (16.7)	0.036	.850
Homocysteine level	15.1 ± 11.2	14.0 ± 9.35	0.428	.670
Median onset to treatment time, min (IQR)	373 (287–471)	393 (311–440)	−0.480	.633
Median onset to imaging time, min (IQR)	357 (270–457)	380 (299–425.5)	−0.476	.636
Median onset to hospital time, min (IQR)	330 (260–445)	370 (287–415)	−0.634	.528
Mean length of HMCAS (mm) (IQR)	21 (18.1–24.8)			
ASPECTS, median (IQR)	5 (1–6)	6 (5–8)	−2.621	.010*
Admission NIHSS score, points, median (IQR)	18 (16–24)	11.5 (9–15)	5.856	<.001*
Discharge NIHSS score, points, median (IQR)	9 (6–19)	5 (3–9.25)	2.585	.012*
3‐month mRS score, grade, median (IQR)	4 (3–6)	2 (1–4)	3.863	<.001*
Mortality 3‐month follow‐up	25 (47.2)	6 (20)	6.043	.014*

Abbreviations: ASPECTS, Alberta Stroke Program Early CT score; HMCAS, hyperdense middle cerebral artery sign; HT, hemorrhagic transformation; ICA, internal carotid artery; IQR, interquartile range; MCA, middle cerebral artery; mRS, modified Rankin Scale; *n*, number; NIHSS, National Institutes of Health Stroke Scale; *SD*, standard deviation; TICA, terminal internal carotid artery.

* Indicates statistical significance.

**TABLE 4:** Baseline factors associated with HT for all patients

Variables	Univariable analysis		Multivariable analysis	
	OR (95% CI)	*p*‐value	OR (95% CI)	*p*‐value
Age	1.014 (0.990; 1.038)	.251		
Gender	1.725 (1.010; 2.946)	.046*	1.599 (0.905; 2.826)	.106
Systolic pressure at admission	1.005 (0.995; 1.015)	.300		
Diastolic pressure at admission	0.997 (0.979; 1.015)	.735		
Glucose value at admission	0.942 (0.834; 1.064)	.340		
Atrial fibrillation	1.554 (0.902; 2.677)	.112		
Homocysteine level at admission	1.006 (0.976; 1.036)	.710		
Admission NIHSS score	1.113 (1.059; 1.170)	<.001*	1.081 (1.026; 1.139)	.003*
HMCAS	3.585 (2.102; 6.114)	<.001*	2.762 (1.571; 4.854)	<0.001*
Antiplatelets	1.027 (0.580; 1.816)	.928		
Anticoagulation	1.359 (0.737; 2.507)	.325		
Antihypertensive	1.033 (0.620; 1.723)	.900		
Anticholesterol	0.820 (0.446; 1.508)	.524		

Abbreviations: ASPECTS, Alberta Stroke Program Early CT score; HMCAS, hyperdense middle cerebral artery sign; NIHSS, National Institutes of Health Stroke Scale.

* Indicates statistical significance.

**TABLE 5:** Baseline factors associated with HT in HMCAS group

Variables	Univariable analysis		Multivariable analysis	
	OR (95% CI)	*p*‐value	OR (95% CI)	*p*‐value
Age	1.048 (1.011; 1.086)	0.010*	1.037 (0.996; 1.079)	0.076
Gender	1.703 (0.800; 3.625)	0.167		
Systolic pressure at admission	1.000 (0.987; 1.014)	0.966		
Diastolic pressure at admission	0.998 (0.971; 1.026)	0.897		
Glucose value at admission	0.949 (0.832; 1.082)	0.431		
Atrial fibrillation	1.619 (0.744; 3.388)	0.201		
Homocysteine level at admission	0.992 (0.957; 1.028)	0.647		
Admission NIHSS score	1.162 (1.079; 1.251)	<0.001*	0.994 (0.888; 1.114)	0.923
Antiplatelets	0.825(0.377; 1.808)	0.632		
Anticoagulation	1.385 (0.593; 3.232)	0.452		
Antihypertensive	0.519 (0.250; 1.078)	0.079		
Anticholesterol	0.698 (0.289; 1.682)	0.423		
Length of HMCAS	1.219 (1.125; 1.321)	<0.001*	1.216 (1.076; 1.374)	0.002*

Abbreviations: ASPECTS, Alberta Stroke Program Early CT score; HMCAS, hyperdense middle cerebral artery sign; NIHSS, National Institutes of Health Stroke Scale.

* Indicates statistical significance.

## 3 RESULT

Of the 289 study patients in this study, 199 were males and 90 were females, with a mean age of 66 ± 11.4 years (range, 22–92). The patients had the following initial signs/symptoms: disturbance of consciousness, dyskinesia, sensory dysfunction, glossolalia, or blurred vision. The interclass correlation analysis between the two observers showed excellent agreement for HMCAS (*κ* value 0.895). The joint evaluation after consensus was agreed that the initial CT showed HMCAS in 121 (41.9%, 121/289) patients and no HCMAS in 168 (58.1%, 168/289) patients.

Differences between the clinical characteristics of the patients (age; sex; history of hypertension, blood pressure at admission; diabetes mellitus; glucose value at admission; prestroke medications) of the HMCAS and non‐HMCAS patients were not significant (*p* > .05). There was a greater incidence of M1 occlusion in HMCAS patients than non‐HMCAS (χ^2^ = 4.738, *p* = .03). HMCAS patients had worse initial stroke severity (median admission NIHSS score, χ^2^ = 5.725, *p *< .001), greater incidence of atrial fibrillation (χ^2^ = 8.918, *p *= .003), higher homocysteine level (χ^2^ = 4.711, *p *= .30), lower ASPECTS score (*t* = −5.835, *p *< .001), higher rate of HT (χ^2^ = 23.128, *p *< .001), higher mRS score (χ^2^ = 5.653, *p *< .001), and lower survival at 3 months (χ^2^ = 16.674, *p *< .001) than in patients without HMCAS (Tables 1 and 2). Interestingly, we also found the homocysteine level at admission in AF patients was higher than patients without AF (*t* = 2.780, *p *= .006).

**FIGURE 1:**
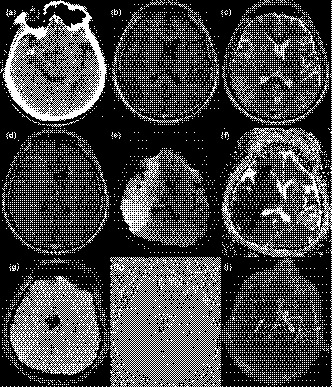
Male, 64 years. (a) CT shows high‐density performance of the right middle cerebral artery on admission at 8 hr from onset of symptoms(arrow). (b–d) T1 weighted imaging(T1WI), T2 weighted imaging(T2WI) and Fluid‐attenuated inversion recovery (FLAIR)imaging shows the right cerebral hemisphere with abnormal signal after admission, about 24 hr since onset of symptoms. (e and f) Diffusion‐weighted imaging (DWI) shows the lesions with high signal, corresponding to apparent diffusion coefficient (ADC) undervalued after admission, about 24 hr since onset of symptoms. (g and h) Susceptibility‐weighted imaging (SWI) shows hemorrhage of the thalamus after admission, about 24 hr since onset of symptoms. (i) Magnetic resonance angiography (MRA) shows occlusions involving the right middle cerebral artery and internal carotid artery (arrow), and double focal stenosis of the anterior cerebral artery, posterior cerebral artery, and left middle cerebral artery focal

The HTs were categorized into 4 different subtypes according to the study by Berger et al. (2001). Hemorrhagic infarction type 1 (HI‐1): small petechiae along the margins of the infarct; HI‐2: more confluent petechiae within the infarcted area, but without space‐occupying effect; Parenchymal hematoma type 1 (PH‐1): hematoma in ≤ 30% of the infarcted area with some slight space‐occupying effect; PH‐2: dense hematoma > 30% of the infarcted area with substantial space‐occupying effect or as any hemorrhagic lesion outside the infarcted area. There were 83 (28.7%, 83/289) patients with secondary HT, including 15 (18.1%, 15/83) patients with hemorrhagic infarction type 1 (HI‐1), 15 (18.1%, 15/83) with HI‐2, 19 (22.9%, 19/83) with parenchymal hematoma type 1 (PH‐1), and 34 (40.9%, 34/83) with PH‐2. PH‐2 occurred significantly more frequently in HMCAS patients than in non‐HMCAS patients (χ^2^ = 3.971, *p *= .046, Table 2, Figure 1).

Table 3 compares the clinical characteristics of HCMAS versus non‐HCMAS patients who developed HT. HMCAS patients with HT were significantly older than non‐HMCAS patients with HT (*t* = 2.473, *p *= .015). The respective incidence of atrial fibrillation and 3‐month follow‐up mortality rates were higher in HMCAS patients with HT than in non‐HMCAS patients with HT (χ^2^ = 3.944, *p *= .047; χ^2^ = 6.043, *p *= .014, respectively). HMCAS Patients with HT had worse initial stroke severity (median admission NIHSS score, χ^2^ = 5.856, *p *< .001), lower ASPECTS score (*t* = −2.621, *p* = .01) and higher mRS score at 3 months (χ^2^ = 3.863, *p* < .001) than those without HMCAS.

**FIGURE 2:**
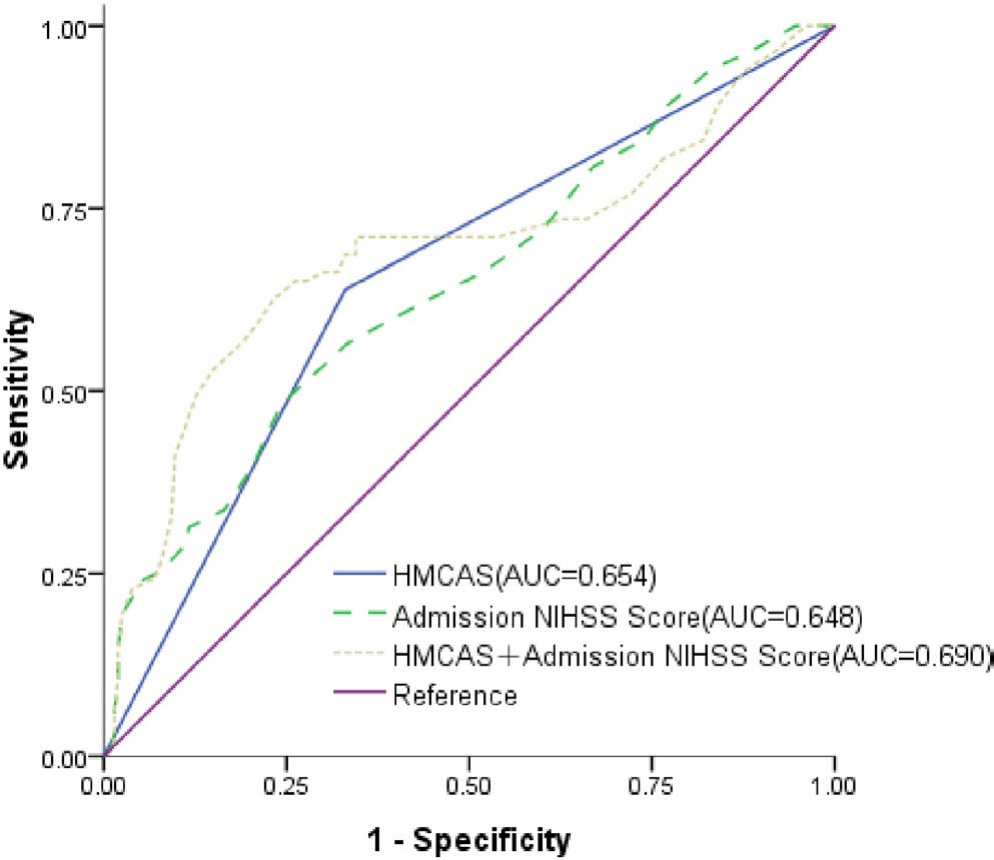
ROC curves comparing the potential of HMCAS to predict HT in large area infarction

Univariate logistic regression analysis was applied for the binary outcomes with HT and without HT. HMCAS was significant in the binary analysis (unadjusted OR = 3.585, 95% CI: 2.102–6.114, *p *< .001). At admission NIHSS score (unadjusted OR = 1.113, 95% CI: 1.059–1.170, *p* < .001) was also significant for HMCAS. Multiple logistic regression analysis identified HT was independently associated with HMCAS (adjusted OR = 2.762, 95% CI: 1.571–4.854, *p* < .001) and admission NIHSS score (adjusted OR = 1.081, 95% CI: 1.026–1.139, *p* = .003) after adjusting for age, gender, blood pressure at admission, glucose value at admission, atrial fibrillation, homocysteine level at admission and prestroke medications (Table 4). To examine the performance of baseline characteristics for predicting HT, ROC curves were developed separately for the two groups, with adjustment for age, gender and other clinical factors as the basic model (Figure 2). HMCAS showed relatively good prediction capabilities for HT (AUC = 0.654), the second was Admission NIHSS score (AUC = 0.648). A combined analysis of HMCAS and Admission NIHSS score showed a fair potential for predicting HT (AUC = 0.690, Figure 2).

In the HMCAS group, multiple logistic regression analysis shows HMCAS with HT was independently associated with length of HMCAS (adjusted OR = 1.216, 95% CI: 1.076–1.374, *p* < .001) after adjusting for the potential confounders (Table 5).

## 4 DISCUSSION

The prevalence of HMCAS in our study patients with large cerebral infarctions was 41.9% (121/289), which is consistent with the results of a previous study (Kummer et al., 1994). HMCAS was first described by Gács in 1983, and was subsequently defined as increased density of the middle cerebral artery, which indicated thrombosis (Riedel et al., 2010). HCMAS has been reported in 17% to 50% of patients with middle cerebral artery infarction, with a specificity of 90%–100% and sensitivity of less than 30% for stroke (Kummer et al., 1994). HMCAS has only been found in 1/3–1/2 cases of DSA‐confirmed thrombosis, which might be accounted for by CT slice thickness. Thrombosis is associated with neurological deficits of increased severity, increased size of the infarction, and worse functional outcome than seen for patients without thrombosis (Man et al., 2015).

In our cohort, HMCAS patients had higher incidence of atrial fibrillation and higher homocysteine level compared to non‐HMCAS patients. Our results indicate that HMCAS and cardiac embolism are related; thus, the early detection and treatment of atrial fibrillation and high homocysteine may prevent the occurrence of related stroke.

Ischemic strokes are often complicated by secondary HT. In our study, the incidence of HT after a large cerebral infarction was 28.7% (83/289), which is consistent with the results of a previous study reporting an incidence of 15%–43% (Jickling et al., 2014). In multiple logistic regression analysis in our study, HT was independently associated with HMCAS and admission NIHSS score after adjusting for age, gender, blood pressure at admission, glucose value at admission, atrial fibrillation, homocysteine level at admission and the other potential confounders.

In Kharitonova et al. (2009) research, HMCAS was not a significant independent risk factor of symptomatic intracerebral hemorrhage using the Stroke‐International Stroke Thrombolysis Register definition (local or remote type 2 parenchymal hemorrhage on the imaging scan 22–36 hr after treatment, accompanied by a decrease in the baseline NIHSS score of ≥ 4 points or death within 24 hr after treatment) (Wahlgren et al., 2007; Wardlaw et al., 2003), but appeared as an independent predictor of symptomatic intracerebral hemorrhage according to the Randomized Controlled Trial definition (any hemorrhage on follow‐up imaging combined with a decrease of at least 1 point on the NIHSS or death before day 7) together with the potential confounders (Kharitonova et al., 2009). Our results were consistent with those of the latter.

At the 3‐month follow‐up period, we observed higher stroke severity in HMCAS patients and less favorable outcomes compared to non‐HMCAS patients, as measured by admission NIHSS score, ASPECTS, and mRS score, and mortality. These results suggest that patients with HMCAS were more likely to experience some degree of neurological deterioration and have infarct‐related HT, including large hematoma, compared to non‐HMCAS patients. The higher incidence of HT may be attributed to the extensive infarcts and severe strokes in HMCAS patients. Presumably due to stroke severity and hence a perceived more clear‐cut need for getting medical care more quickly. The immediate changes after ischemic stroke include ion balance disruption and peri‐infarct depolarization. Followed by change is metabolism failure which consists of an increase of excitotoxicity response, reduced protein synthesis, and disturbed phosphatase activity. Subsequently, free radical release, inflammatory response, apoptosis, necrosis, autophagy, and blood–brain barrier disruption could occur based on the duration and severity of ischemia (Lapchak & Yang, 2017). Berger et al. (2001) pointed out that an infarcted area > 30% (PH‐2) is associated with space‐occupying effects and independently modifies the risk of a worse clinical outcome both early and late after the onset of stroke compared to the clinical outcome of other subtypes of HT. Our results showed that the proportion of PH‐2 was higher in HMCAS patients than in non‐HMCAS patients. It suggested that HMCAS might be a predictor of HT and may also predict the poor clinical outcome of patients with large cerebral infarctions.

Multiple logistic regression analysis showed HT in patients with HMCAS was independently associated with length of HMCAS. The HT we observed in HMCAS patients is likely due to the increased stroke severity as represented by lower ASPECTS, worse NIHSS and 3‐month mRS. HMCAS with long length is generally associated with large cerebral damage. In particular, HMCAS with longer thrombus length highlights the need for clot retrieval in patients with large vessel occlusion. However, HMCAS with a short length does not exclude large cerebral damage (Chrzan et al., 2017; Mair et al., 2016). Thrombus length and estimated occlusion site can be predictors of stroke severity and HT possibility in HMCAS patients. Early recognition of HMCAS may help to identify patients eligible for clot retrieval in timely fashion, although the absence of the HMCAS does not exclude a large vessel occlusion (Chrzan et al., 2017; Puig et al., 2012). Ideally all patients with large vessel occlusive stroke should be considered for clot retrieval.

HT is a common complication of severe stroke and is a manifestation of damage to the blood–brain barrier, loss of microvascular integrity, and disruption of the neurovascular unit (Mair et al., 2016). In our study, HMCAS predicted infarct‐related HT of any type, but the pathophysiology is unclear (Berger et al., 2001). HT may result from augmented collateral circulation into the ischemic zone, perhaps have some relationship with hypertension. Recanalization and distal migration of the thrombus are not the factors that are associated with transformation (Lyden & Zivin, 1993). This could reflect the association with severe stroke. Lindley RI et al. (Lindley et al., 2004) pointed out that severe stroke was a major predictor of HT in patients who did not receive any specific stroke treatment, as well as in those treated with antithrombotic, anticoagulant, or any other drugs. Some studies have pointed out that age, atrial fibrillation, massive cerebral infarction, hypertension, diabetes mellitus, and elevated homocysteine level are risk factors for cerebrovascular disease; and that oral agents (anticoagulant agents and antiplatelet aggregation inhibitors) administered after stroke onset may also have a high risk of vascular rupture and bleeding (Álvarez‐Sabín et al., 2013; Fiolaki et al., 2017). Some studies demonstrated that prophylactic treatment with antiplatelet agents did not lead to a significantly increased risk of HT (Bravo et al., 2008; Diedler et al., 2010). HT and/or malignant cerebral edema are some of the main causes of poor outcome in stroke patients. The mortality of our study patients was 19.2% (60/312). In our study, we think that HT may be related to sclerosis of the small vessels, reperfusion, or change in blood pressure. Treatments such as use of antiplatelet agents that improve the circulation result in changes in the brain microcirculation. Changes caused by reperfusion and increased blood volume in the local circulation increase the possibility of bleeding or aggravate damage to infarcted brain tissue (Campbell et al., 2015; Lansberg et al., 2012; Venturelli et al., 2018).

Our study was a retrospective study that should be interpreted carefully. It was a single‐center study with a relatively small sample, and might have been affected by bias. Further studies are needed to solve these limitations.

We would like to emphasize that HMCAS is the most easily accessible predictor of MCA occlusion, though it is less informative than evidence from angiography. The presence of HMCAS should be considered in clinical decision‐making and risk stratification in acute stroke. Combined with the patient's clinical manifestations, HMCAS on an early CT image can provide a potential target for intervention and aid in the diagnosis of large cerebral infarction, thereby effectively improving patient outcomes.

In conclusion, this study demonstrated that HMCAS is a biomarker that can predict HT and poor outcome of patients with a large cerebral infarction who do not undergo thrombolytic therapy.

## CONFLICT OF INTEREST

The authors declare that there are no conflicts of interest.

## AUTHOR CONTRIBUTION

Benqiang Yang and Yang Duan contributed to the idea of the study; Yang Duan and Jie Hou contributed significantly to analysis and manuscript preparation; Jie Hou and Yu Sun analyzed the data; Jie Hou wrote the manuscript; Libo Zhang, Dengxiang Xing and Xiaoqiu Lee helped perform the analysis with constructive discussions; All authors discussed the results and revised the manuscript.

### Peer Review

The peer review history for this article is available at https://publons.com/publon/10.1002/brb3.2116.

## Data Availability

The data used to support the findings of this study are available from the corresponding author upon request.
